# Chromosome-scale genome assembly of the sea louse *Caligus rogercresseyi* by SMRT sequencing and Hi-C analysis

**DOI:** 10.1038/s41597-021-00842-w

**Published:** 2021-02-11

**Authors:** Cristian Gallardo-Escárate, Valentina Valenzuela-Muñoz, Gustavo Nuñez-Acuña, Diego Valenzuela-Miranda, Ana Teresa Gonçalves, Hugo Escobar-Sepulveda, Ivan Liachko, Bradley Nelson, Steven Roberts, Wesley Warren

**Affiliations:** 1 Interdisciplinary Center for Aquaculture Research, University of Concepción, Concepción, Chile; 2 Laboratory of Biotechnology and Aquatic Genomics, Center of Biotechnology, University of Concepción, Concepción, Chile; 3 Phase Genomics, Inc., Seattle, USA; 4 School of Aquatic and Fishery Sciences (SAFS), University of Washington, Seattle, USA; 5 Bond Life Sciences Center, University of Missouri, Columbia, USA

**Keywords:** Chromosomes, DNA sequencing

## Abstract

*Caligus rogercresseyi*, commonly known as sea louse, is an ectoparasite copepod that impacts the salmon aquaculture in Chile, causing losses of hundreds of million dollars per year. In this study, we report a chromosome-scale assembly of the sea louse (*C. rogercresseyi*) genome based on single-molecule real-time sequencing (SMRT) and proximity ligation (Hi-C) analysis. Coding RNAs and non-coding RNAs, and specifically long non-coding RNAs (lncRNAs) and microRNAs (miRNAs) were identified through whole transcriptome sequencing from different life stages. A total of 23,686 protein-coding genes and 12,558 non-coding RNAs were annotated. In addition, 6,308 lncRNAs and 5,774 miRNAs were found to be transcriptionally active from larvae to adult stages. Taken together, this genomic resource for *C. rogercresseyi* represents a valuable tool to develop sustainable control strategies in the salmon aquaculture industry.

## Abstract (summary)

**Table:** 

Measurement(s)	sequence_assembly • RNA • genome • DNA
Technology Type(s)	sequence assembly process • RNA sequencing • DNA sequencing
Factor Type(s)	developmental stage • sex
Sample Characteristic - Organism	Caligus rogercresseyi
Sample Characteristic - Environment	aquaculture process

Machine-accessible metadata file describing the reported data: 10.6084/m9.figshare.13186865

## Background & Summary

Sea lice are marine copepods that negatively impact the salmon aquaculture worldwide. Two of the most studied sea lice species are *Caligus rogercresseyi* and *Lepeophtheirus salmonis*^1,2^. Annually the salmon farming industry accounts $480 million in losses associated with sea lice, representing 10% of production costs^3–5^. The parasitism on farmed fish causes skin damage, immunosuppression, and co-infection of opportunistic pathogenic bacteria^6–8^. Like all ectoparasites, lice spend a large part of their life cycle on a fish host, displaying specific mechanisms for evading the host’s immune response^9–11^.

The life cycle of lice species is complex and consists of several instars divided by moults. For instance, *C. rogercresseyi* comprises two larval stages (nauplius I, nauplius II and copepodite), four juvenile stages (chalimus I - IV) and one adult stage (female or male)^12^. During the copepodite stage, the process of host identification occurs, preparing the lice for infestation and settlement^8^. The successful infestation process on the host allows the parasite access to nutrients for reproduction and adult development^13,14^. Previous studies have shown that lice have developed physical mechanisms of host recognition. Among these, lice can identify the temperature of the water, salinity changes, and detect the swimming of fish^15^. Host identification via detection of semiochemicals has also been reported^16^. In *C. rogercresseyi*, the presence of advanced chemoreceptors that are capable of identifying specific molecules of different host species has recently been described^17–19^. Herein, the gene family of ionotropic receptors (IRs) are pivotal molecular components for the salmon-louse interaction^20,21^.

Molecular understanding of *C. rogercresseyi* is pivotal to develop sustainable salmon aquaculture. However, genomic resources in this species are limited and poorly characterized at functional levels. In 2012, Yasuike *et al*. (2012) reported a compilation of genomic information on different sea lice genera, including *C. rogercresseyi*. It was not until 2014 that Gallardo-Escárate *et al*.^22^ reported the transcriptome of different life stages during the ontogenetic development as well as differences between male and female adults. This transcriptomic resource served as a basis to identify genes involved in molting, cuticle formation, myogenesis, metabolism, immune response, nervous system development and reproduction. Notably, this gene set has served as a basis for the design of new vaccines^2^.

The increasing availability of transcriptome data has revealed the importance of non-coding RNAs as key regulators of the mRNA transcription^23^. To date, microRNAs (miRNAs) and long non-coding RNAs (lncRNAs) have been studied in several arthropod species with special emphasis on parasitic vectors^24^. Long non-coding RNAs are sequences greater than 200 nucleotides, transcribed in a similar way as coding RNAs^25^. It has been suggested that the number of lncRNAs has increased during evolution, where organisms with more complex mechanisms have acquired more lncRNAs to control diversifying biological processes. MicroRNAs are transcripts around 22 base pairs in length that play an important role in post-transcriptional gene regulation^26^. There are studies that show that miRNAs are not only regulators of biological processes, but can also participate in parasite-host interaction processes^9,27^. In insects affected by viruses, it has been observed that viruses are capable of releasing miRNAs that can regulate the expression of their host genes in order to successfully establish the infection^28^. For *C. rogercresseyi*, several miRNAs expressed during the different stages of development have been characterized^29,30^. Within the profile of miRNAs characterized in *C. rogercresseyi*, the miRNA annotated as Bantam is highly expressed in the infective stage of copepodid. This suggests that Bantam has a key role in the success of the infection. Taken together, these resources reported for the sea louse *C. rogercresseyi* represent a valuable tool to develop sustainable control strategies in the salmon industry. What is lacking is an annotated genome that will facilitate an integrated examination of molecular interactions and provide insight in evolutionary and epigenetics processes that underlie critical life history characteristics. In this study, we report the chromosome-scale whole genome sequence of *C. rogercresseyi* through application of Pacific Biosciences’ single molecule sequencing technique (SMRT) and Phase Genomics’ proximity ligation (Hi-C) based genome scaffolding.

## Methods

### Sample collection, NGS libraries, and sequencing

**Fig. 1:**
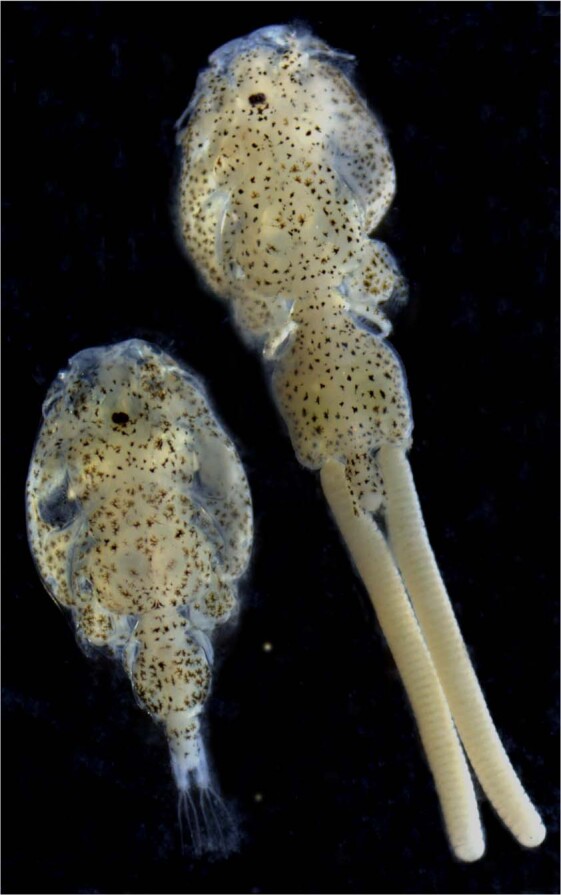
The sea louse Caligus rogercresseyi. Adult female (right) and adult male (left). Magnification 10x.

Adult female specimens of *Caligus rogercresseyi* were collected from Atlantic salmon (*Salmo salar*) at the Caligus Reference Laboratory (CRL), University of Concepción, Chile (Fig. 1). With the aims to reduce the heterozygosity or the number of individuals per pool, female lice were selected for whole-genome sequencing. The samples were frozen in liquid nitrogen to preserve DNA quality, and ten females were used for genomic DNA isolation. High quality DNA was isolated using the Qiagen DNA purification kit (QIAGEN, Germantown, MD, USA) following the manufacturer’s instructions. It is important to note that sea lice are marine copepods exposed to marine environmental conditions and consequently to commensal microorganisms. To reduce bacterial DNA contamination, lice were treated with 20 mg/ml ampicillin (Sigma-Aldrich, USA), 20 mg/ml Kanamycin (US biological, USA), 1x Penicillin-Streptomycin (GIBCO, USA), 100 ug/ml Primocin (Invivogen, USA) for 72 hr prior to the DNA extraction protocol^31^. Furthermore, lice from different developmental stages were separately collected, fixed in RNA Later solution (Ambion, USA), and stored at −80 °C until RNA extractions.

Genomic DNA libraries were constructed according to the manufacturer’s protocols for genome assembly (Table 1). SMRT sequencing yielded 38.32 Gb long reads from 8 SMRT cells (Table 1S). The subreads N50 and average lengths were 11,093 and 6,824 bp, respectively. Hi-C libraries were constructed from whole animals using Phase Genomics’ Animal Hi-C kit and sequenced on an Illumina’s Hiseq4000 platform to yield 238 million of reads. Short-read sequencing libraries were prepared using an insert size of 150 bp obtained from 1 μg of genomic DNA, after fragmentation, end-paired, and ligation to adaptors, respectively. The ligated fragments were fractionated on agarose gels and purified by PCR amplification to produce sequencing libraries.

**Table 1:** Sequencing data generated for sea louse *C. rogercresseyi* genome assembly and annotation.

Library types	Sample	Platform	Molecule	Library size	Data size (Gb)	Application
Long reads	Adult females	PacBio SMRT	DNA	20 kb	38.32	Genome assembly
Hi-C	Adult females	Hiseq4000	DNA	150 bp	35.79	Chromosome construction
Short reads	Developmental stages	MiSeq	RNA	300 bp	52.01	Transcriptome characterization
Short reads	Developmental stages	MiSeq	RNA	150 bp	28.18	miRNome characterization

For transcriptome sequencing, RNA libraries were constructed from nauplius I, nauplius II, copepodid, Chalimus I-II, Chalimus III-IV, males and females, and sequenced by Illumina technology according to the manufacturer’s protocols (Table 1). Briefly, total RNA was extracted from 10 parasites from each stage using the Trizol reagent method (Invitrogen, USA). The quality and integrity of extracted RNAs was measured in a TapeStation 2200 instrument (Agilent, USA), using the R6K Reagent Kit based on manufacturer’s instructions. RNA samples >9 in RIN numbers were selected for library preparation. For whole transcriptome sequencing, 2 μg of total RNA was used for dscDNA libraries with TruSeq Total RNA kit (Illumina, USA). RNA libraries quantification was conducted by qPCR using the NEBNext Library Quant Kit for Illumina (New England Biolabs, USA). The sequencing was performed using the MiSeq platform (Illumina, USA) using a 2 × 250 bp paired-end reads scheme (single flow cell per developmental stage). In addition to generating conventional RNA-seq for 6 developmental stages, small-RNA libraries were also constructed using TruSeq Small RNA Kit (Illumina, USA) for each stage, with libraries run in 41 single-end cycles. Small-RNA libraries were simultaneously sequenced using barcodes according to the manufacturer’s protocols. In total 3 flow cells were used to sequence the 6 developmental stages. A total of 52.01 and 28.18 Gb was yielded for transcriptome and miRNome characterization, respectively (Table 1).

### *De novo* assembly of *C. rogercresseyi* genome

With eight single-molecular real-time cells in the PacBio Sequel platform, we generated 38.32 Gb of high-quality DNA genome information. These long subreads were assembled with the Canu V1.5 package^32^ using default parameters, yielding a draft genome for the sea louse equivalent to 727 Mb with contig N50 of 43,366 bp and 35.55 GC%. The draft genome was assembled with CANU in 25,608 contigs (Table 2). The size genome assembly made by CANU was comparable with previous genome size reported for closely related species^33,34^. However, the manual curation of a subset of contigs revealed bacterial DNA contamination. As we previously mentioned, antibiotic treatment was applied to reduce the natural lice microbiota. However, it appeared that some fraction of the bacterial burden still remained despite the antimicrobial compound used. To reduce the bacterial DNA contamination, all contigs assembled by Canu were firstly filtered against NCBI prokaryotic reference sequence database and then against the reference *C. rogercresseyi* transcriptome (Table 3). For the first filter, BLASTx was applied with an expectation value of 10.0, word size = 3, filter low complexity, protein matrix and gap costs = BLOSUM62, Existence, 11-1, meanwhile that for the second filter a mapping approach was implemented with the following settings using CLC Genomics Workbench V12 (Qiagen, USA): match score = 1, mismatch cost = 2, cost of insertions and deletions = Linear gap cost, insertion cost = 3, deletion cost = 3, length fraction = 0.5, similarity fraction = 0.8, global alignment = No, non-specific match handling = map randomly. Taking advantage of the two filters, we removed all the contigs with a significant match to bacterial DNA, reducing the number of contigs produced by the draft genome for *C. rogercresseyi* made by Canu from 25,608 to 17,711 contigs. Here, the new dataset yielded a draft genome assembly of 519.19 Mb with an N50 of 38,179 bp (Table 4). Notably, the DNA contamination produced by the natural microbiota found in *C. rogercresseyi* was ∼30%. This fact shows the importance of the microbiota in louse biology, revealing putative associations with the pathogenesis of this ectoparasite.

**Table 2:** Genome assembling using PacBio SMRT sequencing in *C. rogercresseyi*.

Label	Assembly statistics
Number of contigs (> = 0 bp)	25,608
Number of contigs (> = 1000 bp)	25,608
Number of contigs (> = 5000 bp)	22,577
Number of contigs (> = 10000 bp)	18,391
Number of contigs (> = 25000 bp)	9,054
Number of contigs (> = 50000 bp)	3,063
Total length (> = 0 bp)	727,321,577
Total length (> = 1000 bp)	727,321,577
Total length (> = 5000 bp)	717,854,023
Total length (> = 10000 bp)	686,707,002
Total length (> = 25000 bp)	529,822,257
Total length (> = 50000 bp)	321,900,412
Number of contigs	25,608
Largest contig	6,415,100
Total length	727,321,577
GC (%)	35.55
N50	43,366
N75	23,550
L50	3,963
L75	9,700
Number of N’s per 100 kbp	0.00

*All statistics are based on contigs of size > = 500 bp, unless otherwise noted (e.g., “# contigs (> = 0 bp)” and “Total length (> = 0 bp)” include all contigs).

**Table 3:** Statistics of transcriptome *de novo* assembly for the sea louse *C. rogercresseyi*.

Transcriptome *de novo* assembly	Statistics
N75	526
N50	1,156
N25	2,548
Minimum (bp)	260
Maximum (bp)	19,659
Average (bp)	1,020
Number of contigs	63,444

**Table 4:** Statistics of genome assembly and Hi-C analysis for the sea louse *C. rogercresseyi*.

Label	Statistics
***PacBio assembly***	
Assembly size	519,118,635
Contig (CTG) N50	38,179
CTGs	17,711
CTGs > 10KB	15,186
CTGs > 5KB	17,318
***Hi-C mapping***	
Total read pairs (RPs) analyzed	238,645,537
High quality (HQ)* RPs	9,24%
HQ RPs > 10KB apart (CTGs > 10KB)	5.32%
Intercontig HQ RPs (CTGs > 10KB)	46.70%
Same strand HQ RPs	7.80%
Split reads	12.55%

### Chromosome assembly of *C. rogercresseyi* using chromatin interaction mapping analysis

**Fig. 2:**
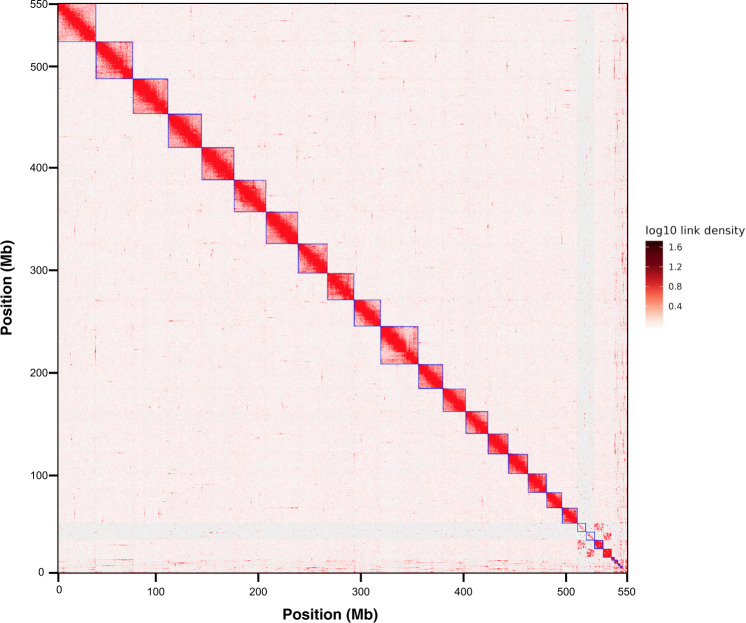
The sea louse Caligus rogercresseyi genome contig contact matrix using Hi-C data. The blue squares represent the draft scaffold. The color bar illuminates the Hi-C contact density in the plot.

**Fig. 3:**
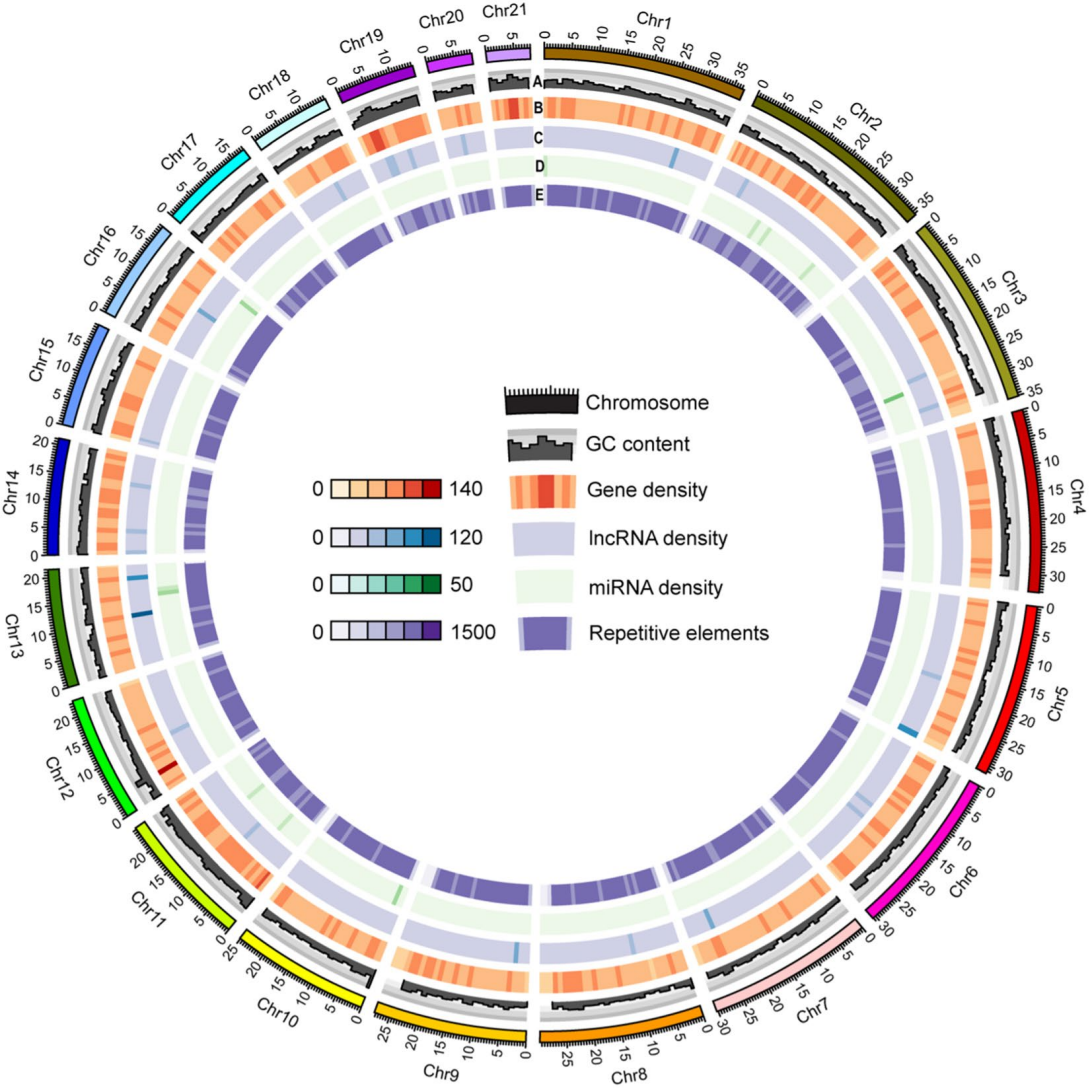
The sea louse Caligus rogercresseyi genome. The circos plot shows the genomic features for the 21 pseudo-chromosomes. A) GC content, B) Gene density, C) lncRNA density, D) miRNA density and E) Repetitive elements. The chromosome size is shown in Mb scale.

In vivo Hi-C is a technique that maps physical DNA-DNA proximity across the entire genome^35,36^. The method was introduced as a genome-wide version of its predecessor, 3 C (Chromosome Conformation Capture)^37^, and has been used as a powerful tool in chromosome-scale genome assembly of many animals in recent years. In this study, Hi-C experiments and data analysis on adult females were used for the chromosome assembly of the sea louse *C. rogercresseyi*. Here, two Hi-C libraries were prepared and sequenced by Phase Genomics (Seattle, WA, USA), resulting in ∼100x coverage and ∼238 million 150-bp paired-end reads (Table 4). The Hi-C analysis evidenced that 46.70% of high-quality reads analysed showed intercontig signals or Cis-close position (<10kbp on the same contig), and an additional 5.32% of sequence reads revealed a Cis-far conformation (>10Kbp on the same contig). To order and orient the 17,711 contigs Hi-C reads were aligned using Bowtie2^38^ and scaffolding performed using Proximo (Phase Genomics, Seattle, WA, USA). We then applied Juicebox^39^ for visual inspection and manual correction. We also manually removed 7,897 scaffolds that were microbe-sized and disconnected from the rest of the assembly. We obtained the first chromosome-level high-quality *C. rogercresseyi* assembly with an N50 scaffold of 29.78 Mb, providing a useful genomic resource for research in sea louse biology and also, to develop novel control strategies applied to the salmon aquaculture (Table 5). In order to visualize the scaffold’s length construction, the *in vivo* Hi-C data were used to generate 21 pseudo-chromosomes assembled with PacBio consensus long DNA reads (Fig. 2). The largest scaffold was assembled from 1,235 contigs, a size of 36.77 Mb. Meanwhile, the smallest scaffold was 7.98 Mb of length and consisted of 396 original contigs (Fig. 3). Notably, the number of contigs in scaffolds were 16,931 (100% of all contigs in chromosome clusters, 95.6% of all contigs) and 505.27 Mb of genome size (100% of all length in chromosome cluster, 97.33% of all sequence length). The completeness of genome assembly was assessed by the single-copy ortholog set (BUSCO, V3.0.2) against Eukaryota, Metazoa, and Arthropoda^40^. The results indicated a complete BUSCO of 78.9% [S:75.3%, D:3.6%] and a fragmented BUSCO of 13.5% [M:13.6%, n:303].

**Table 5:** *De novo* assembly of *C. rogercresseyi* genome using chromatin interaction mapping.

Scaffold number	Number of contigs	Length (bp)
1	1235	36,773,507
2	1097	35,015,117
3	821	33,056,366
4	879	31,368,135
5	947	30,517,993
6	847	30,174,979
7	909	29,781,277
8	941	27,708,916
9	856	25,041,035
10	852	24,730,313
11	1045	35,690,048
12	867	22,871,160
13	775	21,425,356
14	753	21,073,284
15	698	19,021,882
16	708	18,455,137
17	638	17,498,262
18	626	14,617,582
19	629	14,227,552
20	412	8,245,778
21	396	7,983,448
**Total**	**16931**	**505,277,127**
N50		29,781,277

*Number of scaffolds: 21 (100% of all contigs in chromosome clusters, 95.6% of all contigs).

### Repetitive element and non-coding gene annotation in *the C. rogercresseyi* genome

Repetitive elements and non-coding genes in the sea louse genome were annotated by homologous comparison and *ab initio* prediction. RepeatMasker^41^ was used for homologous comparison by searching against the Repbase database and RepeatModeler^42^. According to these analyses, about 269.83 Mb Mb of repeat sequences were annotated, which accounted for 51.9% of the whole genome. Herein, DNA transposons, LINE, and LTR transposable elements were identified (Table 6). Useful genome information for population genetic studies is the identification of simple sequence repeats (SSRs) or microsatellites. The mining of SSRs revealed that the *C. rogercresseyi* genome has 441,494 SSR sequences, where 65.76% represent dinucleotide motifs (Table 7). The total of SSR sequences accounted for 1.39% of the whole genome, and the genome distribution was correlated with the chromosome size (Fig. 1S). Furthermore, SSRs type dinucleotides, and specifically, the motifs AC/GT were the most abundant, representing the 65.76% of the total microsatellite sequences (Fig. 2S). Trinucleotides and tetranucleotides were found in 32.46% of the SSRs sequences (Fig. 3 and Table 7).

**Table 6:** Classification and distribution of repeats based on RepeatModeler from *C. rogercresseyi* genome.

Type	Family	Copy Numbers
DNA Transposon	DNA	1,065
	DNA/Academ	792
	DNA/CMC-Chapaev-3	7,607
	DNA/CMC-Transib	262
	DNA/Ginger	357
	DNA/Merlin	1,907
	DNA/MuLE-MuDR	1,764
	DNA/P	5,656
	DNA/PiggyBac	5,731
	DNA/RC	1,073
	DNA/Sola	482
	DNA/TcMar-Fot1	178
	DNA/TcMar-Mariner	12,717
	DNA/TcMar-Tc1	35,361
	DNA/TcMar-Tc2	121
	DNA/hAT	5,689
	DNA/hAT-Ac	369
	DNA/hAT-Blackjack	108
	DNA/hAT-Charlie	7,186
	DNA/hAT-Tag1	188
	DNA/hAT-Tip100	6,023
	DNA/hAT-Tol2	1,199
	DNA/hAT-hATm	10,502
	DNA/hAT-hATw	365
	DNA/hAT-hATx	2,052
	Total DNA transposons	108,754
LINE	LINE	3,942
	LINE/Jockey	237
	LINE/L1	39,977
	LINE/L1-Tx1	9,089
	LINE/L2	6,853
	LINE/LOA	18,678
	LINE/Penelope	104
	LINE/R1	421
	LINE/R2	1,407
	LINE/RTE-BovB	1,012
	LINE/RTE-X	206
	Total LINE	81,926
	SINE	0
LTR	LTR/Copia	196
	LTR/DIRS	333
	LTR/Gypsy	5,787
	LTR/Pao	932
	Total LTR	7,248
**Total**	**Transposable elements**	**197,928**
	Simple repeats	39,847
	Unknown	585,381

**Table 7:** Simple Sequence Repeats (SSR) of *C. rogercresseyi* genome using SSR Finder analysis.

SSR type	Number of SSR	Size (bp)	Genome coverage* (%)
Dinucleotide	290,331	5,107,439	1.0108
Trinucleotide	61,621	739,346	0.1463
Tetranucleotide	81,696	1,077,404	0.2132
Pentanucleotide	6,677	94,058	0.0186
Hexanuleotide	1,169	24,385	0.0048
Total	441,494	7,042,632	1.3938

*Coverage estimated by the genome size of 505,277,127 bp.

### Protein-coding genes prediction and functional annotation in the *C. rogercresseyi* genome

**Fig. 4:**
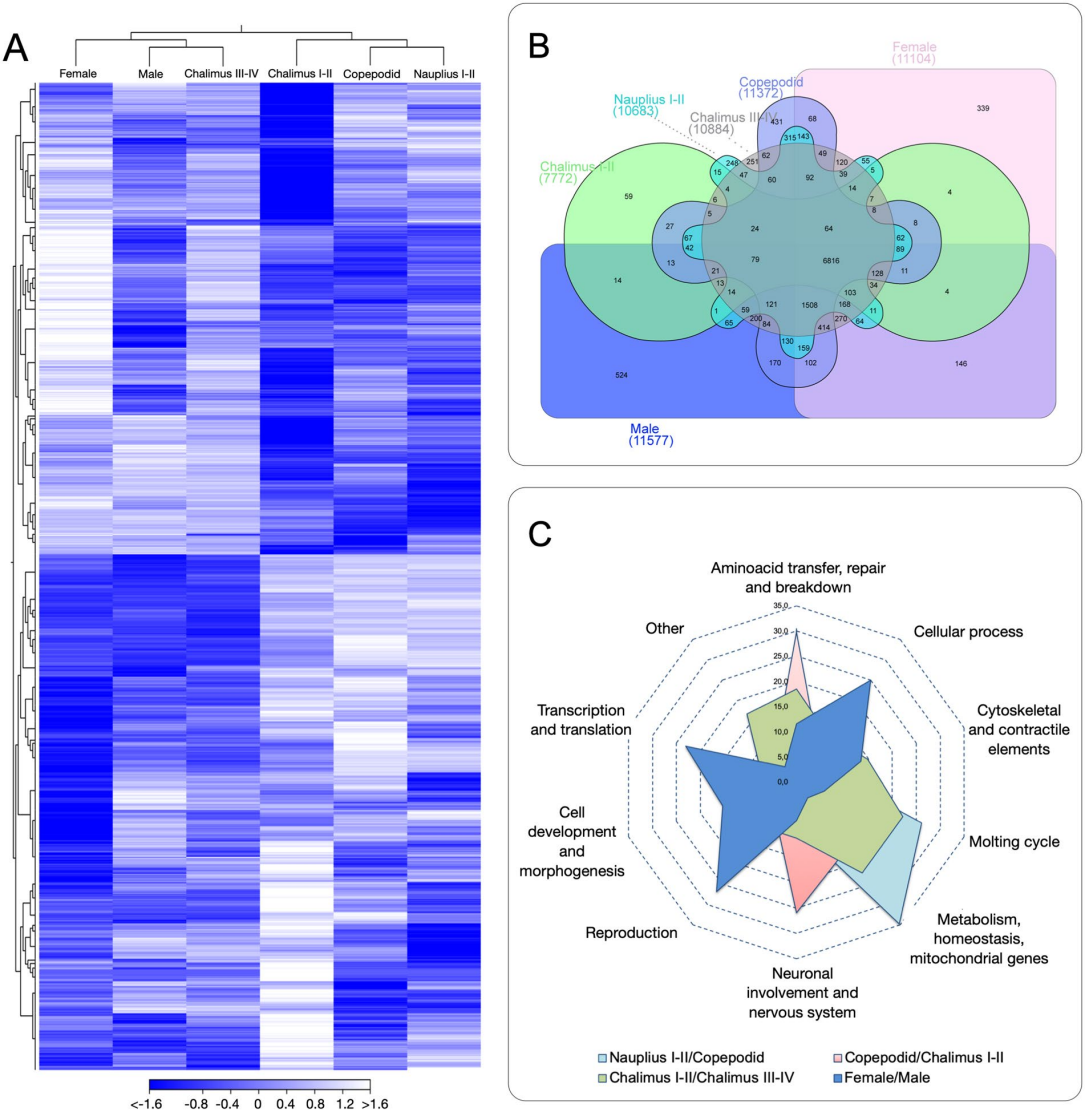
Stage-specific transcriptome analysis in the sea louse Caligus rogercresseyi. (**A**) Transcriptome patterns of coding genes during the lifecycle. The heatmap was based on Transcripts Per Million (TPM) calculation and hierarchical clustering on Manhattan distances with average linkage. White colors mean upregulated coding genes, blue colors downregulated genes. (**B**) Venn diagram showing shared and unique genes expressed among the six developmental stages. (**C**) GO enrichment of stage-specific genes (P-value ≤ 10–16;|fold-change| > 5) annotated for key biological processes differentially expressed. The radar plot represents the comparison between two developmental stages according the C. rogercresseyi lifecycle.

For the identification of protein-coding genes, two approaches were employed for the sea louse genome, including homologous comparison and *ab initio* prediction. For homologous comparison, the protein sequences from *Caenorhabditis elegans* (GCA_000002985.3), *Drosophila melanogaster* (GCA_000001215.4), and *Daphnia pulex* (GL732539.1) genomes were extracted using the respectively published genomes. and aligned against the sea louse genome using TBLASTN (e-value < 1e-5). Gene sequence structure of each candidate genes was predicted using GeneWise^43^. For *ad initio* prediction, five tools were used to predict protein-coding genes using the Genome Sequence Annotation Server “GenSAS” (https://www.gensas.org)^44^. Specifically, Augustus, Braker, GeneMarkES, SNAP, and GlimmerM were used with default parameters. Finally, a non-redundant reference gene set was generated using EvidenceModeler (EVM) and PASA2 tools^45^. Taken together 25,510 protein-coding genes were identified. (Fig. 3 and Table 8). Additionally, 437 tRNAs were predicted using tRNAscan-SE, and 39 rRNA genes were annotated using RNAmmer via GenSAS. For non-coding RNAs with putative regulatory roles, 5,774 miRNAs and 6,308 long-ncRNAs were identified and annotated within the *C. rogercresseyi* genome using transcriptome sequencing data (Fig. 4 and Table 9). For functional annotation, the predicted proteins within the sea louse genome were searched by homology against four databases of InterPro^46^, GO^47^, KEGG KO^48^, and Swissprot^49^. Overall, 88.05%, 68.85%, 64.02%, and 91.02% of genes matched entries in these databases, respectively. A total of 23,686 genes (93%) were successfully annotated by gene function and conserved protein motifs (Table 10).

**Table 8:** Prediction of protein-coding genes in the sea louse *Caligus rogercresseyi* genome.

Gene set		Gene number	Average gene length (bp)	Ave. CDS length (bp)	% of genome
*Homologous comparison*	*Caenorhabditis elegans*	16,827	19,740	758	0.66
	*Drosophila melanogaster*	21,767	18,970	934	0.86
	*Daphnia pulex*	32,143	28,760	1,475	1.69
*Ab initio*	Augustus	26,970	16,870	887	4.72
	Braker	71,522	20,380	695	9.91
	GeneMarkES	82,621	28,890	1,177	19.26
	SNAP	35,139	14,740	612	10.25
	GlimmerM	32,588	22,960	172	1.91
EvidenceModeler		25,663	26,210	1,572	2.91
Official gene set (PASA refinement)		23,686	25,510	1,518	2.43

**Table 9:** Summary of non-coding RNA annotation in the sea louse *Caligus rogercresseyi*.

Type	Number	Average length (bp)	Total length (bp)	% of genome
miRNA	5,774	21,97	126.831	0.0251
lncRNA	6,308	520.38	3.282.545	0.6474
tRNA	437	74.20	29,230	0.0069
rRNA (28-18 s; 5 s)	39	639.51	24,941	0.0049

**Table 10:** Statistics for genome annotation of the sea louse *Caligus rogercresseyi*.

Database	Number	Percent
InterPro	20,856	88.05
GO	16,308	68.85
KEGG KO	15,165	64.02
Swissprot	21,676	91.51
NR	22,814	96.31
Total	23,686	

*e-value threshold of the 1e-5 was applied during the homolog searching for the functional annotation.

## Technical Validation

### RNA integrity

Before constructing RNA-seq libraries, the concentration and quality of total RNA were evaluated using Agilent 2100 Bioanalyser (Agilent, USA). Three metrics, including total amount, RNA integrity, and rRNA ratio, were used to estimate the content, quality, and degradation level of RNA samples. In this study, only total RNAs with a total amount of ≥10 μg, RNA integrity number ≥8, and rRNA ratio ≥1.5 were finally subjected to construct the sequencing library.

### Quality filtering of Illumina sequencing raw reads

The initial raw sequencing reads were evaluated in terms of the average quality score at each position, GC content distribution, quality distribution, base composition, and other metrics. Furthermore, the sequencing reads with low quality were also filtered out before the genome assembly and annotation of gene structure.

**Table 11:** Software and URLs.

Tool	Website
BUSCO	https://busco.ezlab.org/
RepeatMasker	http://www.repeatmasker.org/
RepeatModeler	http://www.repeatmasker.org/
AUGUSTUS	http://bioinf.uni-greifswald.de/augustus/
BRAKER2	http://exon.gatech.edu/GeneMark/braker1.html
GeneMark-ES	http://exon.gatech.edu/GeneMark/
Genscan	http://genes.mit.edu/GENSCAN.html
GlimmerM	http://www.cbcb.umd.edu/software/glimmerm/
SNAP	https://github.com/KorfLab/SNAP
BLAST+	http://blast.ncbi.nlm.nih.gov/Blast.cgi?PAGE_TYPE=BlastDocs&DOC_TYPE=Download
BLAT	https://genome.ucsc.edu/FAQ/FAQblat.html; https://github.com/icebert/pblat
Diamond	https://github.com/bbuchfink/diamond
PASA	http://pasapipeline.github.io/
getorf	http://emboss.sourceforge.net/apps/release/6.3/emboss/apps/getorf.html
RNAmmer	http://www.cbs.dtu.dk/services/RNAmmer/
SSR Finder	GenSAS custom tool, MainLab Bioinformatics
tRNAScan-SE	http://lowelab.ucsc.edu/tRNAscan-SE/
EVidenceModeler	http://evidencemodeler.github.io/
InterProScan	http://www.ebi.ac.uk/Tools/pfa/iprscan5/

## Data Records

DNA and RNA sequencing runs were deposited to NCBI Sequence Read Archive (SRA)^50–52^. The assembled genome has been deposited at NCBI assembly with the accession number ASM1338718v1^53^. Additional files containing repeated sequences, gene structure, and functional prediction were deposited in the *Figshare* database^54^.

## Supplementary information

Table 1S

Supplementary Figures

## Data Availability

The sequence data were generates using the Genome Sequence Annotation Server “GenSAS” (https://www.gensas.org)^44^. No custom computer codes were generated in this work (Table 11).
