# Supplementary material for: Chromosome-scale genome assembly of the sea louse Caligus rogercresseyi by SMRT sequencing and Hi-C analysis
Source: Sci Data. 2021 Feb 11;8:60. doi: 10.1038/s41597-021-00842-w (PMC7878743; doi:10.1038/s41597-021-00842-w)
Supplement: Supplementary file 1 — Table 1S [file 41597_2021_842_MOESM1_ESM.docx]

**Table 1S.** Statistics of the PacBio SEQUEL sequencing data from *C. rogercresseyi*

|  | SMRT Cell 1 | SMRT Cell 2 | SMRT Cell 3 | SMRT Cell 4 | SMRT Cell 5 | SMRT Cell 6 | SMRT Cell 7 | SMRT Cell 8 |
| --- | --- | --- | --- | --- | --- | --- | --- | --- |
| Total Bases (Gb) | 5.0 | 5.23 | 4.6 | 5.2 | 4.75 | 4.31 | 4.29 | 4.94 |
| Polymerase RL Mean (bp) | 7,636 | 8,601 | 7,093 | 7,197 | 7,402 | 7,208 | 5,742 | 6,576 |
| Polymerase N50 (bp) | 14,250 | 15,750 | 13,250 | 13,750 | 13,750 | 13,250 | 11,250 | 12,250 |
| Insert Length Mean (bp) | 7,278 | 8,049 | 6,768 | 6,801 | 7,032 | 6,872 | 5,536 | 6,289 |
| Insert N50 (bp) | 13,250 | 14,750 | 12,250 | 12,750 | 12,750 | 12,750 | 10,750 | 11,750 |
