# Supplementary material for: Chromosome-scale genome assembly of the sea louse Caligus rogercresseyi by SMRT sequencing and Hi-C analysis
Source: Sci Data. 2021 Feb 11;8:60. doi: 10.1038/s41597-021-00842-w (PMC7878743; doi:10.1038/s41597-021-00842-w)
Supplement: Supplementary file 2 — Supplementary Figures [file 41597_2021_842_MOESM2_ESM.pdf]

Supplementary figures

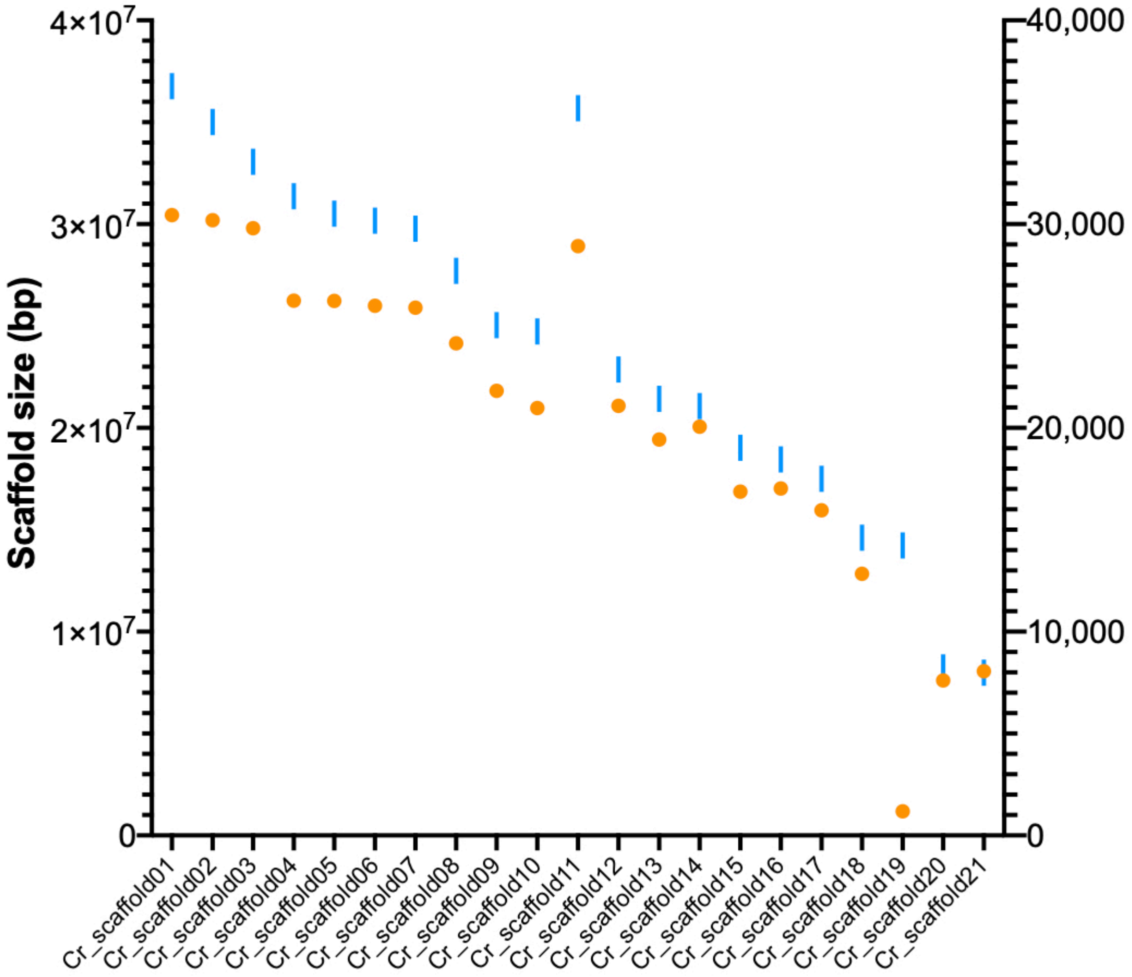

Figure 1S. Distribution of Simple Sequence Repeats (SSR) identified by SSR Finder. SSRs were annotated through the 21 pseudo-chromosomes with a total number of 441.494 sequences. Vertical blue lines and orange dots represent the scaffold size (bp) and the number of SSRs, respectively.

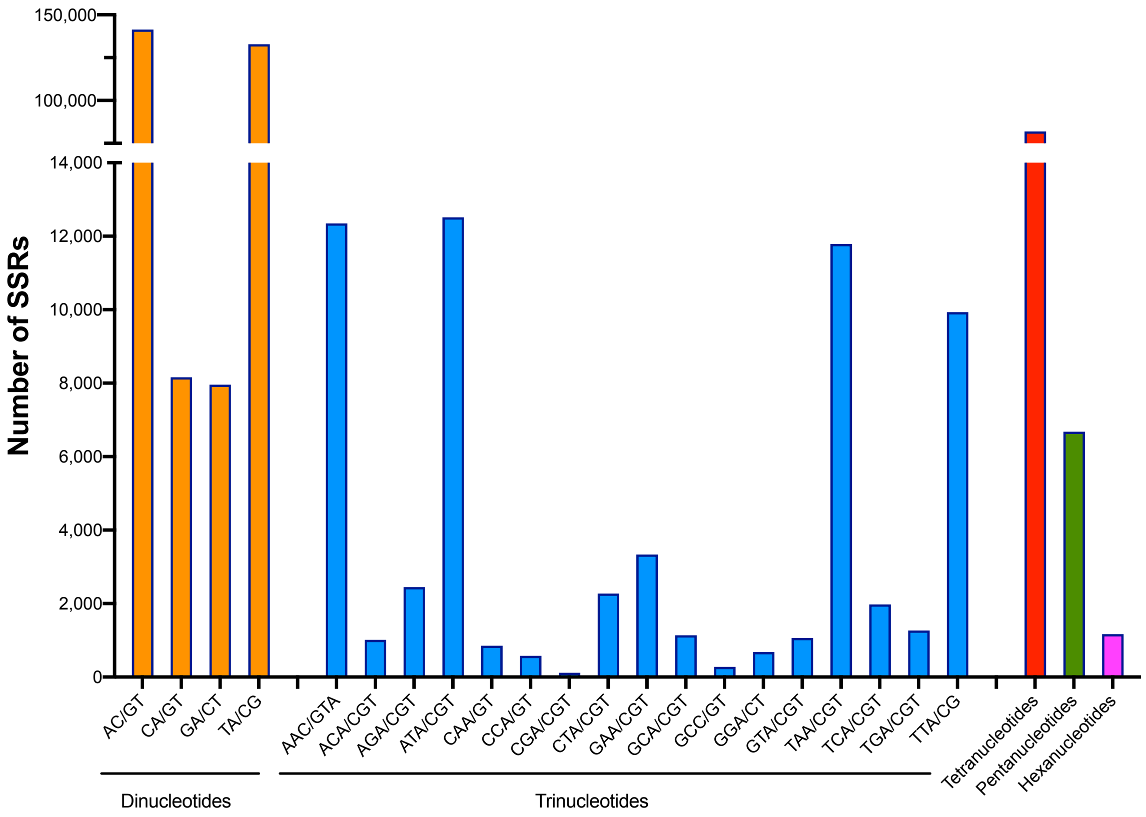

Figure 2S. Simple Sequence Repeats (SSR) types identified in the sea louse *C. rogercresseyi* genome.
